# Incidental findings during lung low‐dose computed tomography cancer screening in Australia and Canada, 2016–21: a prospective observational study

**DOI:** 10.5694/mja2.52649

**Published:** 2025-05-04

**Authors:** Asha Bonney, Diane M Pascoe, Mark W McCusker, Daniel Steinfort, Henry Marshall, Annette McWilliams, Fraser J Brims, Emily Stone, Paul Fogarty, Jeremy D Silver, Brad Milner, Elizabeth Silverstone, Eugene Hsu, Duy Nguyen, Christopher Rofe, Cameron White, XinXin Hu, John Mayo, Renelle Myers, Kwun M Fong, Renee Manser, Stephen Lam

**Affiliations:** ^1^ Royal Melbourne Hospital Melbourne VIC; ^2^ The University of Melbourne Melbourne VIC; ^3^ The Prince Charles Hospital Brisbane QLD; ^4^ Thoracic Research Centre, the University of Queensland Brisbane QLD; ^5^ Fiona Stanley Hospital Perth WA; ^6^ The University of Western Australia Perth WA.; ^7^ Sir Charles Gairdner Hospital Perth WA; ^8^ Curtin Medical School, Curtin University Perth WA; ^9^ St Vincent's Hospital Sydney NSW; ^10^ St Vincent's Clinical School, University of New South Wales Sydney NSW; ^11^ Epworth Eastern Hospital Melbourne VIC; ^12^ Statistical Consulting Centre, the University of Melbourne Melbourne VIC; ^13^ Vancouver General Hospital Vancouver Canada; ^14^ The University of British Columbia Vancouver Canada; ^15^ BC Cancer Vancouver Canada; ^16^ UQ Thoracic Research Centre, the Prince Charles Hospital Brisbane QLD

**Keywords:** Mass screening, Lung diseases, Early detection of cancer, Computed tomography

## Abstract

**Objectives:**

To investigate the type and frequency of incidental findings in people at high risk of lung cancer who undergo baseline low‐dose computed tomography (LDCT) lung cancer screening in Australia and Canada.

**Study design:**

Prospective observational study; sub‐study of the single‐arm International Lung Screen Trial (ILST) lung cancer screening study.

**Setting, participants:**

Australian and Canadian people enrolled in the ILST, 25 August 2016 – 21 November 2020; inclusion criteria: aged 50–80 years, active smoking history, and high risk of lung cancer (estimated six‐year lung cancer risk of 1.51% or more, based on the PLCO_m2012_ risk prediction model; or a smoking history of 30 pack‐years or more). Initial LDCT screening was undertaken at one of five participating hospitals in Australia and one in Canada.

**Main outcome measures:**

Prevalence of incidental findings during baseline LDCT lung cancer screening (using a research checklist), by country, classified by experienced radiologists as requiring or not requiring clinical follow‐up; reporting of incidental findings in clinical reports for treating physicians (two Australian sites only).

**Results:**

A total of 4403 participants completed baseline LDCT screening at the six participating hospitals. The mean age (64–65 years) and the proportions of participants who currently smoked (47–55%) were similar at all six sites; the proportion of female participants was larger in Sydney (52%) and Vancouver (51%) than the other sites (39–44%). At least one incidental finding was made during baseline LDCT screening of 3225 people (72.8%); findings in 454 people (10.3%) required clinical follow‐up. The most frequent incidental findings were coronary artery calcification (3022 of 4380 participants with recorded results, 69.0%) and emphysema (2378 of 4401, 54.0%). Marked differences between the Australian and Canadian sites in the prevalence of incidental findings were noted, and also between the two Australian sites in their communication of incidental findings in clinical screening reports.

**Conclusion:**

Incidental findings during lung cancer screening were frequent, and clinical reporting of these findings was inconsistent. When LDCT lung cancer screening is introduced in Australia, a standardised reporting template should be used to provide clear guidance about the clinical significance of such findings.

**Trial registration:**

ClinicalTrials.gov, NCT02871856 (prospective, 18 August 2016).

**The known**: Lung cancer screening with low‐dose computed tomography (LDCT) is associated with lower lung cancer‐related mortality among people at high risk of the disease.

**The new**: Incidental findings in 73% of Australian and Canadian participants in an LDCT lung cancer screening trial included findings requiring clinical attention in 10% of people. The reporting of incidental findings in clinical reports for treating physicians was inconsistent.

**The implications**: The new Australian lung cancer screening program should include standardised reporting of incidental findings, as they have consequences for the wellbeing of screening participants and the cost‐effectiveness of the screening program.

Lung cancer is the leading cause of cancer‐related death in Australia and overseas.^1^ Screening for lung cancer using low‐dose computed tomography (LDCT) reduces lung cancer‐related mortality in people at particular risk of lung cancer with an active tobacco smoking history.^2^, ^3^, ^4^ The Australian Department of Health and Aged Care plans to commence a national LDCT lung cancer screening program by mid‐2025.^5^

Incidental findings are a major consideration for lung cancer screening using LDCT. Unlike other cancer screening techniques, it can detect tissue changes other than lung cancer and pulmonary nodules. The largest relevant lung cancer screening randomised controlled trial to date, the United States National Lung Screening Trial, reported that significant incidental findings were identified in 33.8% of people in the LDCT group, most frequently emphysema (43% of participants) and coronary artery calcification (12%).^6^

Some incidental findings could facilitate earlier detection of clinically significant changes, such as coronary artery calcification and cardiovascular disease, and vertebral fractures and osteoporosis. However, LDCT findings of thyroid nodules,^7^ for example, could lead to unnecessary investigations and consequently increase the harms and costs of lung cancer screening. In Europe and the United States, guidelines include recommendations about which incidental findings are clinically relevant, and which are probably not.^8^, ^9^, ^10^

The prevalence of incidental findings in American^6^ and European lung cancer screening cohorts^11^ has been reported, but not for an Australian or Canadian lung cancer screening trial cohort. We therefore prospectively investigated the type and frequency of incidental findings in people at high risk of lung cancer who underwent LDCT screening in Australia and Canada.

## Methods

The International Lung Screen Trial (ILST) is a multisite single arm LDCT lung cancer screening study; its primary objectives are to compare lung cancer screening participant selection criteria and to evaluate a nodule management protocol.^12^ The target sample size was 4000 participants from Australia (five sites, selected by geographic location and local expertise) and Canada (one site, selected for its available infrastructure and expertise);^12^ the overall target was subsequently increased after the study commenced because greater resources allowed the addition of ILST sites in Hong Kong, the United Kingdom, and Spain.^13^, ^14^ The ILST was registered with the United States Clinical Trials Registry on 18 August 2016 (clinicaltrials.gov: NCT02871856); recruitment commenced in Australia and Canada on 25 August 2016 and concluded on 21 November 2020.

Participants for the ILST were recruited through social media, primary care providers, government mailouts, news and radio advertising, and personal referrals from friends and family.^13^ People were eligible to participate if they were aged 55–80 years, had an active smoking history, an Eastern Cooperative Oncology Group (ECOG) performance status of 0 or 1, and either an estimated six‐year lung cancer risk of 1.51% or more, based on the PLCO_m2012_ risk prediction model, or a smoking history of 30 pack‐years or more (US Preventive Task Force 2013 criterion).^15^ The PLCO_m2012_ is a logistic regression model that incorporates age, race or ethnic group, education level, body mass index, history of chronic obstructive pulmonary disease (COPD), family history of lung cancer, personal history of cancer, and smoking history to estimate the risk of lung cancer;^16^ the US Preventive Task Force 2013 criteria, in contrast, are based solely on age and smoking history.^15^ The PLCO_m2012_ has been validated in Australia and Canadian cohorts of people with active tobacco smoking histories.^17^

### Baseline low‐dose computed tomography lung cancer screening

The prospective observational study reported in this article, nested within the ILST, included all Australian (Brisbane: Prince Charles Hospital; Melbourne: [I] Royal Melbourne Hospital and [II] Epworth Box Hill Hospital; Perth: Fiona Stanley Hospital; Sydney: St Vincent's Hospital) and Canadian (Vancouver General Hospital) ILST sites. The first baseline LDCT screens were undertaken in August 2016, and the final baseline LDCT screens in July 2021. We report our study in accordance with the Strengthening the Reporting of Observational Studies in Epidemiology (STROBE) guidelines.^18^

The primary aim of the study was to determine the prevalence of incidental findings in baseline LDCT screens in an Australian and Canadian lung cancer screening sample. The secondary aim was to describe the pattern of clinical reporting of LDCT lung cancer screening in Australia, based on a subset of ILST participants.

All participants underwent baseline LDCT screening for lung cancer; further interval scans and investigations for lung cancer were undertaken according to the trial protocol, using the PanCan nodule calculator score.^12^ LDCT (120 kV tube voltage, 40–50 mA tube current) was performed without intravenous contrast in the supine position during a single inspiratory breath hold. LDCT reporting was completed by experienced chest radiologists (at least three hundred computed tomography [CT] chest readings during the past three years) using a standardised research template.

Age, sex, smoking status, smoking history (in pack‐years), ethnic background, other medical conditions, medications, and spirometry findings were collected at baseline in the health questionnaire and assessment for all enrolled ILST participants.

### Incidental findings

In addition to nodules and findings relevant to lung cancer, information on incidental findings unrelated to lung cancer was collected using a checklist. Incidental findings were categorised as present or absent for emphysema, interstitial lung abnormalities, airway abnormalities (mucous impaction, bronchial wall thickening, bronchiectasis, bronchiolectasis), and coronary artery calcification; other incidental findings — pleural, cardiovascular, gastrointestinal, endocrine, musculoskeletal, breast, and lymph node findings — were recorded as actionable (requiring clinical follow‐up) or non‐actionable. If the reporting radiologist did not select an option for an incidental finding in the research checklist, it was treated as missing information and the participant was excluded from the summary statistic for the finding. The total number of participants with complete information for each incidental finding included in the checklist was recorded. The research checklist was a data collection tool for the ILST and was not provided to participants or treating clinicians. ILST radiologists instead provided a clinical report for each participant's treating clinicians, which also specified lung cancer risk and recommended screening follow‐up. Classification and reporting of incidental findings were at the radiologists’ discretion, and there was no pre‐specified method or template for reporting incidental findings in LDCT clinical reports. A consensus guide to incidental findings definitions, including their categorisation, is included in the Supporting Information, table 1.

To assess the communication of incidental findings to clinicians, all incidental findings in Brisbane and Melbourne (I) baseline LDCT screening clinical reports were manually extracted by a clinician (Melbourne [I]: author AB; Brisbane: author HM) and classified as requiring action if included in the conclusion of the clinical report, or as not requiring action if included only in the body of the report or not at all. Retrospective review of baseline LDCT clinical reports was limited to these two sites for resource reasons.

### Statistical analysis

Statistical analyses were performed in R 4.2.1 (R Foundation for Statistical Computing). We summarise parametric data as means with standard deviations (SDs) and non‐parametric data as medians with interquartile ranges (IQRs). The statistical significance of between‐group differences were assessed in Pearson χ^2^ and Fisher exact tests (categorical variables); *P* < 0.05 was deemed statistically significant.

### Ethics approval

This study was approved by the Prince Charles Hospital human research ethics committee (HREC/16/QPCH/181); all relevant local governance approvals were obtained. All study participants provided written informed consent to participation in the study.

## Results

A total of 4403 participants were enrolled and completed baseline LDCT screening at the six participating hospitals (Box 1). The mean age (64–65 years) and the proportions of participants who currently smoked (47–55%) were similar at all six sites; the proportion of female participants was larger in Sydney (52%) and Vancouver (51%) than at the other sites (39–44%). Median smoking history exceeded 40 pack‐years at all sites; it was slightly higher at Australian sites than in Vancouver, where median PLCO_m2012_ scores were also slightly higher. Most participants were classified as having European ethnic backgrounds (86–97% by site); the proportion of Indigenous participants was slightly higher in Brisbane (2.4%) and Vancouver (2.3%) than at the other sites (0–1.6%) (Box 2). No baseline medical conditions were reported by 2928 participants (67%) (Supporting Information, table 2).

Box 1 **Participant selection for International Lung Screen Trial (ILST) substudy of incidental findings during low‐dose computed tomography lung cancer screening in Australia (five sites) and Canada (one site), 2016–2021**

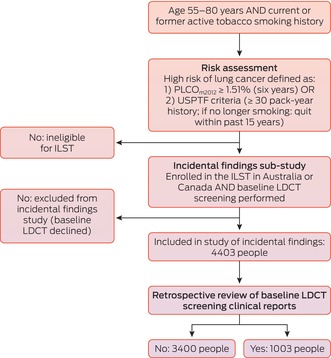

LDCT = low‐dose computed tomography; USPTF = United States Preventive Task Force.

Box 2 **Baseline characteristics of participants in the International Lung Screen Trial substudy of incidental findings during low‐dose computed tomography lung cancer screening in Australia (five sites) and Canada (one site), 2016–2021**

**Table:** 

Characteristic	Brisbane	Melbourne (I)	Melbourne (II)	Perth	Sydney	Vancouver
Number of participants	595	408	127	591	378	2304
Age (years), mean (SD)	64 (6.4)	64 (6.1)	65 (6.5)	65 (6.5)	65 (6.1)	64 (6.4)
Sex						
Women	235 (39%)	167 (41%)	56 (44%)	242 (41%)	196 (52%)	1179 (51%)
Men	360 (61%)	241 (59%)	71 (56%)	349 (59%)	182 (48%)	1125 (49%)
Currently smokes tobacco	281 (47%)	225 (55%)	69 (54%)	287 (49%)	193 (51%)	1095 (48%)
Smoking history (pack‐years), median (IQR)	44 (35–58)	46 (36–57)	43 (34–54)	43 (34–54)	42 (34–55)	40 (33–49)
PLCO_m2012_ score, median (IQR)	2.9 (1.9–4.8)	3.1 (1.9–5.1)	2.7 (1.7–4.9)	2.7 (1.7–4.9)	3.1 (2.0–5.3)	2.6 (1.7–4.3)
Ethnic background						
African	2 (0.3%)	1 (0.5%)	0	1 (0.2%)	1 (0.3%)	27 (1.2%)
Asian	2 (0.3%)	8 (2.0%)	4 (3.1%)	8 (1.4%)	2 (0.5%)	225 (9.8%)
European	576 (97%)	391 (96%)	113 (89%)	570 (96%)	365 (96%)	1984 (86%)
Indigenous	14 (2.4%)	3 (0.7%)	0	3 (0.5%)	6 (1.6%)	53 (2.3%)
Other	1 (0.2%)	5 (1.2%)	10 (7.9%)	9 (1.5%)	4 (1.1%)	15 (0.7%)
Education (highest level)						
Year 8 or lower	221 (37%)	130 (32%)	33 (26%)	217 (37%)	108 (29%)	248 (11%)
Year 8 to 11	86 (14%)	80 (20%)	26 (20%)	118 (20%)	67 (18%)	564 (24%)
High school graduate	108 (18%)	57 (14%)	20 (16%)	109 (18%)	84 (22%)	316 (14%)
Vocational certificate	77 (13%)	43 (11%)	8 (6%)	58 (10%)	20 (5.3%)	569 (25%)
Some university/college	66 (11%)	54 (13%)	26 (20%)	67 (11%)	61 (16%)	403 (17%)
University graduate	37 (6%)	42 (10%)	14 (11%)	22 (4%)	38 (10%)	204 (9%)
Unknown	0	2 (0.5%)	0	0	0	0
Spirometry						
FEV_1_ (% predicted), median (IQR)	87% (85–88%)	94% (92–95%)	90% (86–93%)	90% (88–92%)	87% (85–89%)	88% (87–89%)
Forced expiratory ratio, median (IQR)	66% (66–67%)	70% (69–71%)	71% (70–73%)	69% (68–69%)	73% (72–74%)	70% (70–71%)

FEV_1_ = forced expiratory volume in one second; IQR = interquartile range; SD = standard deviation.

### Prevalence of incidental findings at baseline LDCT screening

At least one incidental finding was made during baseline LDCT screening of 3225 people (72.8%); findings in 454 people (10.3%) required clinical follow‐up or action, including 351 of 2099 Australian (16.7%) and 103 of 2304 Canadian participants (4.5%). Data completeness varied by research checklist incidental finding type (Box 3).

Box 3 **Incidental findings in the International Lung Screen Trial substudy of incidental findings during low‐dose computed tomography lung cancer screening in Australia (five sites) and Canada (one site), 2016–2021, by country**

**Table:** 

Finding type	Australia	Canada	P*	Missing data (Australia/Canada)
Total number of people	2099	2304		
Pleura			< 0.001	6/0
Actionable	22/2093 (1.1%)	5/2304 (0.2%)		
Non‐actionable	166/2093 (7.9%)	55/2304 (2.4%)		
Normal	1905/2093 (91.0%)	2244/2304 (97.4%)		
Interstitial lung abnormality			< 0.001	129/0
Actionable	101/1970 (6.3%)	2/2304 (0.1%)		
Non‐actionable	18/1970 (1.1%)	66/2304 (2.9%)		
Normal	1497/1970 (92.6%)	2236/2304 (97.0%)		
Airways			< 0.001	20/30
Abnormal	683/2079 (32.9%)	534/2274 (23.5%)		
Normal	1396/2079 (67.1%)	1740/2274 (76.5%)		
Emphysema			< 0.001	2/0
Present	1186/2097 (57.0%)	1192/2304 (51.7%)		
Absent	911/2097 (43.4%)	1112/2304 (48.3%)		
Emphysema (by grade)			—	—
Trivial (< 5%)	407/1186 (34.3%)	834/1192 (70.0%)		
Mild (5–25%)	518/1186 (43.7%)	244/1192 (20.5%)		
Moderate (> 25% to 50%)	186/1186 (15.7%)	80/1192 (6.7%)		
Marked (> 50% to 75%)	52/1186 (4.4%)	25/1192 (2.1%)		
Severe (> 75%)	12/1186 (1.0%)	7/1192 (0.6%)		
Not recorded	11/1186	2/1192		
Coronary artery calcification			< 0.001	19/4
Present	1396/2080 (67.1%)	1626/2300 (70.7%)		
Absent	684/2080 (32.9%)	674/2300 (29.3%)		
Coronary artery calcification (by grade)			—	—
Mild	803/2080 (38.6%)	846/2300 (36.8%)		
Moderate	425/2080 (20.4%)	334/2300 (14.5%)		
Severe	168/2080 (8.1%)	446/2300 (19.4%)		
Cardiovascular (excluding coronary artery calcification)			< 0.001	6/2
Actionable	140/2093 (6.7%)	19/2302 (0.8%)		
Non‐actionable	308/2093 (14.7%)	204/2302 (8.9%)		
Normal	1645/2093 (78.6%)	2079/2302 (90.3%)		
Gastrointestinal			< 0.001	4/2
Actionable	81/2095 (3.9%)	24/2302 (1.0%)		
Non‐actionable	376/2095 (17.9%)	629/2302 (27.3%)		
Normal	1638/2095 (78.2%)	1649/2302 (71.6%)		
Endocrine			< 0.001	9/1
Actionable	73/2090 (3.5%)	32/2303 (1.4%)		
Non‐actionable	79/2090 (3.8%)	97/2303 (4.2%)		
Normal	1938/2090 (97.7%)	2174/2303 (94.4%)		
Musculoskeletal			< 0.001	7/1
Actionable	32/2092 (1.5%)	6/2303 (0.3%)		
Non‐actionable	425/2092 (20.3%)	180/2303 (7.8%)		
Normal	1635/2092 (78.2%)	2117/2303 (91.9%)		
Vertebral			< 0.001	125/3
Actionable	150/1974 (7.6%)	4/2301 (0.2%)		
Non‐actionable	23/1974 (1.2%)	248/2301 (10.8%)		
Normal	1801/1974 (91.2%)	2049/2301 (89.0%)		
Breast			0.15	6/2
Actionable	29/2093 (1.4%)	14/2302 (0.6%)		
Non‐actionable	44/2093 (2.1%)	90/2302 (3.9%)		
Normal	2020/2093 (96.5%)	2198/2302 (95.5%)		
Lymph nodes (axillary and abdominal)			< 0.001	5/2
Actionable	35/2094 (1.7%)	8/2302 (0.3%)		
Non‐actionable	58/2094 (2.8%)	29/2302 (1.3%)		
Normal	2001/2094 (95.6%)	2265/2302 (98.4%)		
Other			< 0.001	31/2
Actionable	76/2068 (3.7%)	43/2302 (1.9%)		
Non‐actionable	161/2068 (7.8%)	780/2302 (33.9%)		
Normal	1831/2068 (88.5%)	1479/2302 (64.2%)		

* Fisher exact test.

Of the 2378 participants with incidental findings of radiological emphysema (54.0%), 1563 (65.7%) reported no history of obstructive airways disease. Ninety‐one of 362 participants with incidental findings of moderate to severe emphysema (25.1%) reported a history of emphysema, including seven of nineteen with incidental findings of severe emphysema. Two of 541 participants with incidental findings of interstitial lung abnormalities reported a history of pulmonary fibrosis.

Among the 3022 participants with incidental findings of coronary artery calcification (69.0%), 94 of 1649 with mild calcification (5.7%), 96 of 759 with moderate calcification (12.6%), and 159 of 614 (25.9%) with severe coronary artery calcification reported histories of coronary artery disease.

Eighteen of 105 people with incidental endocrine findings requiring action (17%) and 30 of 176 with incidental endocrine findings that did not require follow‐up (17%) reported histories of thyroid disorder. Histories of osteoporosis or osteopenia were reported by 24 of 154 people with incidental vertebral findings that required action (15.6%) and 50 of 271 people with incidental vertebral findings that did not require follow‐up (18.5%).

### Clinical reporting of baseline LDCT screening incidental findings

As the baseline reports for all site participants from the Brisbane and Melbourne (I) sites were included in the retrospective review of baseline LDCT clinical reports, there were no missing data (Box 4; Supporting Information, table 3).

Box 4 Incidental findings reported in screening clinical reports for participants screened at the Brisbane or Melbourne (I) sites*

**Table:** 

Finding type	Brisbane	Melbourne (I)	*P* ^†^
Total number of people	595	408	
Emphysema			< 0.001
Actionable	64 (10.8%)	5 (1.2%)	
Non‐actionable	268 (45.0%)	233 (57.1%)	
Not reported^‡^	21 (3.5%)	27 (6.6%)	
Pulmonary fibrosis			0.23
Actionable	10 (1.7%)	2 (0.5%)	
Non‐actionable	25 (4.2%)	17 (4.2%)	
Interstitial lung abnormality			0.21
Actionable	24 (4.0%)	21 (5.1%)	
Non‐actionable	7 (1.2%)	10 (2.5%)	
Bronchiectasis			0.006
Actionable	23 (3.9%)	10 (2.5%)	
Non‐actionable	41 (6.9%)	51 (12.5%)	
Not reported^‡^	99 (16.7%)	72 (17.6%)	
Bronchial wall thickening			0.002
Actionable	21 (3.5%)	2 (0.5%)	
Non‐actionable	51 (8.6%)	49 (12.0%)	
Pleura			< 0.001
Actionable	25 (4.2%)	10 (2.5%)	
Non‐actionable	51 (8.6%)	12 (2.9%)	
Coronary artery calcification			< 0.001
Actionable	173 (29.1%)	43 (10.6%)	
Non‐actionable	245 (41.2%)	212 (52.1%)	
Not reported^‡^	8 (1.3%)	24 (5.9%)	
Valves			< 0.001
Actionable	7 (1.2%)	0	
Non‐actionable	42 (7.1%)	3 (0.7%)	
Pericardium			0.005
Actionable	0	7 (1.7%)	
Non‐actionable	0	0	
Biliary			0.005
Actionable	3 (0.5%)	4 (1.0%)	
Non‐actionable	34 (5.7%)	7 (1.7%)	
Liver			0.011
Actionable	20 (3.4%)	15 (3.7%)	
Non‐actionable	34 (5.7%)	44 (10.8%)	
Pancreas			0.07
Actionable	1 (0.2%)	5 (1.2%)	
Non‐actionable	2 (0.3%)	3 (0.7%)	
Spleen			0.82
Actionable	0	0	
Non‐actionable	8 (1.3%)	4 (1.0%)	
Renal			
Actionable	5 (0.8%)	16 (3.9%)	0.002
Non‐actionable	20 (3.4%)	19 (4.7%)	
Adrenal			0.008
Actionable	4 (0.7%)	5 (1.2%)	
Non‐actionable	1 (0.2%)	8 (2.0%)	
Thyroid			< 0.001
Actionable	9 (1.5%)	20 (4.9%)	
Non‐actionable	8 (1.3%)	20 (4.9%)	
Not reported^‡^	6 (1.0%)	10 (2.5%)	
Diaphragm			0.36
Actionable	3 (0.5%)	0	
Non‐actionable	12 (2.0%)	8 (2.0%)	
Osteopenia			< 0.001
Actionable	7 (1.2%)	1 (0.2%)	
Non‐actionable	62 (10.4%)	7 (1.7%)	
Vertebral fractures			0.27
Actionable	16 (2.7%)	5 (1.2%)	
Non‐actionable	55 (9.2%)	36 (8.8%)	
Not reported^‡^	33 (5.5%)	10 (2.5%)	
Breast			0.08
Actionable	13 (2.2%)	2 (0.5%)	
Non‐actionable	11 (1.8%)	6 (1.5%)	
Lymph nodes: mediastinal/hilar			0.022
Actionable	15 (2.5%)	7 (1.7%)	
Non‐actionable	19 (3.2%)	9 (2.2%)	

* As participants without the incidental findings are not included in this table, there were no missing data.

† Fisher exact test, for actionable and non‐actionable findings in clinical reports.

‡ Incidental findings in research checklist not mentioned in clinical reports.

#### Respiratory system findings

Emphysema was mentioned in LDCT clinical reports as a finding requiring action for 69 of 570 participants at the two sites with incidental findings of radiological emphysema (12%); twelve of these participants (17%) reported a history of COPD. Increasing severity of emphysema was significantly associated with being reported in the conclusion of Brisbane clinical reports (*P* < 0.001) (Supporting Information, table 4). For Melbourne (I), emphysema requiring action was reported in five of 408 clinical LDCT reports; the one incidental finding of severe emphysema was not reported as requiring action. Forty‐eight of 618 incidental emphysema findings (7.8%) were not mentioned in LDCT clinical reports.

None of the 45 participants with actionable incidental findings of interstitial lung abnormalities had known pulmonary fibrosis at enrolment; five reported prior pneumonia.

Incidental findings of bronchiectasis were not mentioned in the clinical reports for 171 of 296 people.

#### Incidental findings of coronary artery calcification

For 32 of 705 participants with incidental findings of coronary artery calcification (4.7%), this finding was not mentioned in their LDCT clinical reports, including fourteen with findings of moderate to severe coronary artery calcification; incidental findings of severe coronary artery calcification were mentioned in the report conclusion for 33 of 71 people (46%). Larger proportions of findings of moderate (93 of 197, 47.2%) or severe coronary artery calcification (33 of 71, 46.5%) were reported in report conclusions than of mild calcification (85 of 413, 20.6%) (Supporting Information, table 5).

#### Other system findings

Incidental findings of thyroid abnormalities were reported in the LDCT clinical report conclusion for all 29 people with incidental findings deemed actionable, nineteen of whom did not report histories of thyroid disease.

Ninety‐four of 113 participants with incidental liver findings did not have histories of liver disease, including three of 35 participants with incidental liver findings requiring action.

Five of 60 participants with incidental renal abnormality findings (8%) reported histories of renal disease.

Incidental findings of vertebral fractures 43 of 155 people (27.7%) were not mentioned in LDCT reports.

## Discussion

We report the first investigation of incidental findings in LDCT lung cancer screening of a large cohort of participants in Australia and Canada. Incidental findings were frequent (72.8% of participants); incidental findings requiring clinical follow‐up were made for 16.7% of participants in Australia. The most frequent findings were coronary artery calcification (69.0% of participants) and emphysema (54.0%). which is unsurprising for people at high risk of respiratory disease with substantial tobacco smoking histories. Coronary artery calcification was also the most frequent incidental finding in a London cohort of people at high risk of lung cancer screened using LDCT (64.2% of 11 115 participants).^19^ Clinically significant incidental findings were reported for 33.8% of 26 455 LDCT screening participants in the United States National Lung Screening Trial, most frequently emphysema (43.0%).^6^

The marked differences between the Australian and Canadian sites in the prevalence of incidental findings could be related to differences in population characteristics or in radiology reporting practices. Study participants were selected because they were at high risk of lung cancer, and large proportions of participants at all sites currently smoked, but the median smoking history at Australian sites was greater than for Vancouver. All Canadian LDCT screening reports were prepared by a single experienced chest radiologist, the Australian reports by several experienced chest radiologists. Even within Australia, however, the reporting of certain incidental findings as requiring action (potentially clinically significant) in clinical reports for treating clinicians differed between the two sites examined; for example, coronary artery calcification requiring follow‐up was reported in a larger proportion of LDCT clinical reports from Brisbane (29.1%) than of those from Melbourne (I) (10.6%).

Our study was conducted under real world conditions, and our findings illustrate the potential for variation in recommendations and care when there is no clear and consistent guidance about reporting. The Yale Lung Screening and Nodule Program has proposed structured LDCT screening reports, incorporating not only a description of potentially significant incidental findings (S classification of the Lung Imaging Reporting and Data System, Lung–RADS), but also, when relevant, a findings summary and guideline‐based management recommendations in the report conclusion.^10^ Similar recommendations proposed in Europe^9^ recognise the possibility of inappropriate investigation and management of incidental findings. It is important for lung cancer screening programs that radiologists uniformly characterise and describe incidental findings, and that they provide consistent recommendations regarding the need for further assessment in a structured format. Not including some clinically insignificant incidental findings in the LDCT report could reduce the risk of unnecessary investigations. It is also critical that clinicians and screening participants are aware that incidental findings are possible during LDCT lung cancer screening.

In our study, the level of missing research checklist data ranged from two participants (0.05%) with no recorded emphysema outcome to 129 participants (2.9%) without recorded interstitial lung abnormality status. The reporting of incidental findings was inconsistent in an observational study of 37 908 people in 43 United States facilities even after the addition of the S category to Lung–RADS for potentially clinically significant incidental findings; it was used in 0.1% to 37.4% of initial LDCT reports, depending on the site.^20^ Differences between narrative lung cancer screening LDCT reports and synoptic reporting in Canada have been described, with at least one omission noted in 70% of narrative reports.^21^ This suggests that the quality of LDCT lung cancer screening reports should be reviewed and benchmarked across screening sites to ensure that programs provide optimal care.

Most participants with incidental findings in our study did not report relevant clinical histories; for example, fewer than 1% of people with incidental interstitial lung abnormality findings reported pulmonary fibrosis. At a single site in the United States National Lung Screening Trial, incidental interstitial lung abnormality findings were recorded for 9.7% of participants (and was associated with current active tobacco smoking); equivocal lung parenchymal changes were incidental findings in a further 11.5%.^22^ The European joint statement on the management of incidental findings recommended reporting all interstitial lung abnormality findings and further surveillance, or referring the person to a specialist if more than 5% of a whole lung or zone was involved.^9^ The Fleischner Society similarly recommends clinical assessment before deciding on further management.^23^ The workload for primary care providers is increased if clear guidance and streamlined processes are not available. In the Manchester Lung Health Check pilot screening study, the initial protocol for notifying primary care providers about additional findings and recommended management was modified to reduce the burden on primary care, and the lung cancer screening team assumed responsibility for any specialist referrals.^11^

Although inappropriate investigation of incidental findings could adversely affect screening participants and the cost‐effectiveness of LDCT lung cancer screening, clinically significant incidental findings could also improve health care. We have previously discussed, for example, incorporating incidental coronary artery calcification findings into cardiovascular disease risk assessment and care optimisation.^24^ The second most frequent incidental finding in our study was emphysema, found in more than half of the participants, if mostly trivial or mild in severity. Most people with incidental findings of moderate to severe emphysema reported no history of the condition, and lung cancer screening could be an opportunity for earlier detection and intervention in people who have not reported COPD symptoms.^25^ The European statement on the management of incidental findings recommends smoking cessation for all people with incidental emphysema findings, and clinical assessment of those in whom it is moderate to severe.^9^ The American College of Radiology, however, recommends primary care assessment, regardless of severity, and consideration of referral to a respiratory medicine specialist.^8^ Local guidance that takes health care infrastructure and resources into account is important. A recent survey of primary care providers in the United States found that adherence to guidelines for the management of incidental findings was higher when LDCT reports used a standardised category S template (83.3%) than when they did not (51.7%).^26^ This finding was consistent with another that including radiologist recommendations in the LDCT report body (odds ratio [OR], 4.67; 95% confidence interval [CI], 2.23–9.76) and conclusion (OR, 2.58; 95% CI, 1.28–5.18) each increased the likelihood of the incidental finding being investigated further.^27^

### Limitations

The ILST did not provide strict guidance regarding which incidental findings did or did not require clinical follow‐up, and decisions about reporting them were at the discretion of the radiologist, reflecting usual practice. This situation probably contributed to variations in reporting by the participating radiologists. A large volume of information was collected with the LDCT screening research template, including several fields for incidental findings. Missing data may reflect the effort required to complete a comprehensive LDCT lung cancer screening report, which includes incidental findings as well as lung cancer risk; this factor will be important when structuring LDCT screening reports for the Australian lung cancer screening program. There were some minor discrepancies between incidental findings recorded in the research template and those recorded in narrative clinical reports; they may reflect errors at the time of reporting or differences in recording findings for research and clinical purposes. Further, the ILST commenced before most overseas recommendations for managing incidental findings had been published. The marked differences between the Australian and Canadian sites in the proportions of incidental findings deemed to require clinical follow‐up could be related to cultural or population differences, or to a single radiologist reporting all findings for the one Canadian site. Resource limitations meant that incidental finding reporting could only be reviewed for LDCT reports from two Australian sites. The results of multiple independent statistical tests should be interpreted with caution, given the increasing risk of type 1 errors with multiple comparisons.

As the large majority of ILST participants had European ethnic backgrounds, our findings must be generalised to people with other ethnic backgrounds with caution. The participating sites may not necessarily be representative of their respective countries. As Aboriginal and Torres Strait Islander Australians are at greater risk of lung cancer and lung cancer‐related mortality than other Australians,^28^ major health benefits could be achieved by a successful lung cancer screening program for Indigenous Australians.^29^ However, more information about incidental finding rates in Aboriginal and Torres Strait Islander people is needed; the Australian Lung Screen Trial, which recently commenced recruitment, will investigate lung cancer screening of Indigenous people living in rural and remote communities.^30^

Information about other medical conditions and medications were based on participant responses to baseline questionnaires. Participants may have not reported known conditions or been unaware of some conditions, which could mitigate the clinical significance of some incidental findings. We did not assess subsequent investigations or the management of incidental findings to evaluate their impact on lung cancer screening participants, including investigation harms and costs. Five‐year follow‐up outcomes, including subsequent investigations and costs of screening for Australian ILST participants, will be reported as they become available.

### Conclusion

Incidental findings were recorded during LDCT lung cancer screening for 72.8% of Australian and Canadian participants aged 55–80 years at high risk of lung cancer, including clinically significant findings in 10.3% of participants. Our findings will be a useful reference for lung cancer screening programs in the two countries. When lung cancer screening commences in Australia, the judicious reporting of incidental findings could increase the benefits and reduce the harms of the program. All screening sites should consider structured reporting of incidental findings and provide clear guidelines regarding their management according to local standards of care. LDCT lung cancer screening reduces lung cancer‐related mortality among people at particular risk of the disease, but the impact of incidental findings on outcomes should be investigated, including complications of investigations, participant distress, and program cost‐effectiveness.

## Open access

Open access publishing facilitated by the University of Melbourne, as part of the Wiley – the University of Melbourne agreement via the Council of Australian University Librarians.

## Competing interests

No relevant disclosures.

## Data sharing

The study data can be accessed by contacting the corresponding author.

## Supporting information


Supplementary methods and results
