# Supplementary material for: Incidental findings during lung low‐dose computed tomography cancer screening in Australia and Canada, 2016–21: a prospective observational study
Source: Med J Aust. 2025 May 4;222(8):403–11. doi: 10.5694/mja2.52649 (PMC12050252; doi:10.5694/mja2.52649)
Supplement: Supplementary file 1 — Supplementary methods and results [file MJA2-222-403-s001.pdf]

MJA
The Medical Journal of Australia

Supporting Information

Supplementary methods and results

This appendix was part of the submitted manuscript and has been peer reviewed. It is posted as supplied by the authors.

Appendix to: Bonney A, Pascoe DM, McCusker MW, et al. Incidental findings during lung low-dose computed tomography cancer screening in Australia and Canada, 2016–21: a prospective observational study. *Med J Aust* 2025; doi: 10.5694/mja2.52649

Supplementary methods

Table 1. Classification of incidental findings.

		**Not actionable**	**Actionable***
**Pulmonary**	Emphysema	Can describe the dominant pattern (centrilobular, panlobular, paraseptal).	
	Interstitial lung disease	No evidence of interstitial lung abnormality.<br>Mild interstitial lung abnormality.	Established interstitial lung disease.
	Airways disease: bronchiectasis/ airway abnormalities		Presence of increased broncho arterial ratio, lack of airway tapering, mucus impaction, bronchial wall thickening.
	Pulmonary artery		Main pulmonary artery dilatation ≥2.8cm (females) or ≥3cm (males) or diameter greater than the ascending aorta.
**Cardiovascular**	Coronary artery calcification	Absence of coronary artery - calcification based on simple visual assessment.<br>Mild = isolated flecks of coronary artery calcification (may be actionable pending clinical context).	Moderate = coronary artery calcification in between criteria of mild or heavy.<br>Severe = continuous coronary artery calcification in segments.
	Cardiovascular		Aortic valve calcification via visual assessment.<br>Pericardial effusion (volume ≥50ml), pericardial mass, pericardial thickening.
	Aorta		Aneurysm (>5cm for ascending aorta, >4cm for descending aorta).
**Endocrine**	Thyroid	Absence of suspicious features and below size criteria.	Suspicious features such as local invasion, abnormal lymph nodes or meeting size criteria.
	Breast		Breast lesions are actionable as breast on CT can be difficult to interpret and require correlation with screening mammography or ultrasound.
	Adrenal	Benign features (calcified, ≤ 10 Hounsfield Units).	Indeterminate features.
**Gastrointestinal**	Liver	Benign imaging features e.g. cysts.	All other liver lesions, ultrasound recommended.<br>Fatty liver disease at the discretion of the reporting radiologist.
	Oesophagus	Hiatus hernia.	Increased oesophageal thickness or mass.<br>Dilated oesophagus.
**Genitourinary**	Renal	Benign cysts.	Inconclusive renal lesions, heterogenous lesions, ultrasound recommended.<br>All other lesions.
**Musculoskeletal**	Osteoporosis		Osteopenia, osteoporosis.
	Diaphragm		Diaphragmatic hernia.<br>Elevated hemidiaphragm.
**Lymph nodes**		Short axis <10mm or benign features.	Short axis ≥10mm.

* Final definition of an “actionable” finding was at the discretion of the reporting radiologist.

Supplementary results

Table 2. Reported medical conditions and medications at baseline

Characteristic	Australia	Canada	*P**
Number of participants	2099	2304	
**Medical conditions**			
**No medical conditions**	1522 (72.5%)	1406 (61.0%)	
**Cardiovascular**			
Coronary artery disease	156 (9.0%)	109 (4.8%)	<0.001
Angina	63 (3.6%)	71 (3.1%)	0.41
Myocardial infarct	108 (6.1%)	95 (4.2%)	0.005
Hyperlipidaemia	757 (43.3%)	858 (38.6%)	0.003
Hypertension	670 (37.9%)	798 (35.3%)	0.10
Peripheral vascular disease	43 (2.4%)	34 (1.5%)	0.035
Stroke	61 (3.5%)	72 (3.2%)	0.61
Heart failure	8 (0.5%)	12 (0.5%)	0.73
Valvular heart disease	29 (1.6%)	33 (1.5%)	0.64
Arrythmia	162 (9.3%)	248 (11.1%)	0.06
**Respiratory**			
Asthma	233 (13.2%)	276 (12.3%)	0.40
Chronic obstructive pulmonary disease	203 (9.7%)	447 (19.4%)	<0.001
Emphysema	259 (12.4%)	121 (5.3%)	<0.001
Chronic bronchitis	162 (7.7%)	217 (9.4%)	0.46
Pneumonia	268 (15.2%)	614 (27.3%)	<0.001
Pulmonary fibrosis	3 (0.2%)	6 (0.3%)	0.53
**Endocrine**			
Diabetes	200 (11.3%)	272 (12.0%)	0.48
Thyroid disease	172 (9.8%)	308 (13.6%)	<0.001
Osteoporosis/ osteopenia	180 (10.3%)	277 (12.3%)	0.046
**Infections**			
Human immunodeficiency virus/acquired immunodeficiency syndrome	10 (0.6%)	25 (1.1%)	0.08
Tuberculosis	14 (0.8%)	43 (1.9%)	0.004
**Liver disease**	128 (7.2%)	183 (8.1%)	0.28
**Renal disease**	54 (3.0%)	65 (2.9%)	0.75
**Medications**			
**No medications**	558 (28.2%)	576 (25.3%)	0.038
Aspirin	431 (24.4%)	708 (31.2%)	<0.001
Inhaled corticosteroids	109 (6.3%)	248 (11.0%)	<0.001
Bronchodilators	325 (18.4%)	354 (15.7%)	0.024
Prednisolone	69 (4.0%)	77 (3.4%)	0.35
Statin	646 (37.1%)	612 (27.1%)	<0.001

* Fisher exact test.

Table 3. Other incidental findings in clinical reports, by site*

	Brisbane	Melbourne (I)	*P*†
Number of people	595	408	
Respiratory			
**Airways (other)**			<0.001
Actionable	20 (3.4%)	4 (1.0%)	
Non-actionable	54 (9.1%)	4 (1.0%)	
**Atelectasis**			0.23
Actionable	11 (1.9%)	4 (1.0%)	
Non-actionable	114 (19.2%)	66 (16.2%)	
**Pulmonary granulomata**			<0.001
Actionable	18 (3.0%)	20 (4.9%)	
Non-actionable	142 (23.9%)	152 (37.3%)	
**Lung parenchyma (not otherwise specified)**			<0.001
Actionable	42 (7.1%)	7 (1.7%)	
Non-actionable	147 (24.7%)	48 (11.8%)	
Gastrointestinal			
**Oesophagus**			0.90
Actionable	3 (0.5%)	1 (0.2%)	
Non-actionable	0	0	
**Stomach**			0.22
Actionable	2 (0.3%)	0	
Non-actionable	27 (4.5%)	26 (6.4%)	
**Colon**			0.29
Actionable	1 (0.2%)	0	
Non-actionable	8 (1.3%)	2 (0.5%)	
Genitourinary			
**Renal**			0.002
Actionable	5 (0.8%)	16 (3.9%)	
Non-actionable	20 (3.4%)	19 (4.7%)	
Endocrine			
**Adrenal**			0.008
Actionable	4 (0.7%)	5 (1.2%)	
Non-actionable	1 (0.2%)	8 (2.0%)	
**Thyroid**			<0.001
Actionable	9 (1.5%)	20 (4.9%)	
Non-actionable	8 (1.3%)	20 (4.9%)	
Musculoskeletal			
**Chest wall**			0.06
Actionable	2 (0.3%)	1 (0.2%)	
Non-actionable	8 (1.3%)	0	
**Abdominal wall**			0.34
Actionable	0	1 (0.2%)	
Non-actionable	1 (0.2%)	0	
**Spine**			0.08

	**Brisbane**	**Melbourne (I)**	*P*†
Actionable	6 (1.0%)	3 (0.7%)	
Non-actionable	47 (7.9%)	18 (4.4%)	
**Vertebral disc**			<0.001
Actionable	2 (0.3%)	0	
Non-actionable	66 (11.1%)	4 (1.0%)	
**Musculoskeletal: ribs**			0.14
Actionable	0	2 (0.5%)	
Non-actionable	18 (3.0%)	8 (2.0%)	
**Musculoskeletal: degenerative joint disease**			0.004
Actionable	1 (0.2%)	1 (0.2%)	
Non-actionable	51 (8.6%)	14 (3.4%)	
**Bones (not otherwise specified)**			<0.001
Actionable	0	4 (1.0%)	
Non-actionable	4 (0.7%)	12 (2.9%)	
Breast			0.08
Actionable	13 (2.2%)	2 (0.5%)	
Non-actionable	11 (1.8%)	6 (1.5%)	
Lymph nodes			
**Lymph nodes: intrapulmonary**			0.022
Actionable	11 (1.8%)	0	
Non-actionable	36 (6.1%)	25 (6.1%)	
**Lymph nodes-axilla**			0.046
Actionable	2 (0.3%)	0	
Non-actionable	5 (0.8%)	0	
**Lymph nodes- abdominal**			0.66
Actionable	3 (0.5%)	2 (0.5%)	
Non-actionable	1 (0.2%)	2 (0.5%)	
Vascular			
**Aorta**			<0.001
Actionable	18 (3.0%)	2 (0.5%)	
Non-actionable	78 (13.1%)	5 (1.2%)	
**Pulmonary**			0.87
Actionable	6 (1.0%)	3 (0.7%)	
Non-actionable	2 (0.3%)	1 (0.2%)	
**Abdominal**			<0.001
Actionable	0	0	
Non-actionable	1 (0.2%)	0	
Soft tissue			0.24
Actionable	2 (0.3%)	3 (0.7%)	
Non-actionable	3 (0.5%)	0	

* Numbers of participants without the incidental findings are not included in this table. There were no missing data.

† Fisher exact test.

Table 4. Emphysema by grade, by site, as described in clinical reports*

Finding	Brisbane	Melbourne (I)
Total number of people	595	408
**Emphysema**		
Absent†		
Actionable	0	0
Non-actionable	0	21
Not reported	0	0
Severity not recorded†		
Actionable	0	1
Non-actionable	1	0
Not reported	1	0
Trivial†		
Actionable	4	0
Non-actionable	95	89
Not reported	11	16
Mild†		
Actionable	27	0
Non-actionable	121	76
Not reported	9	7
Moderate†		
Actionable	24	3
Non-actionable	37	33
Not reported	0	4
Marked†		
Actionable	7	1
Non-actionable	12	13
Not reported	0	0
Severe†		
Actionable	2	0
Non-actionable	2	1
Not reported	0	0

* There were no missing data.

† As categorised in the research checklist.

Association between emphysema severity and reporting in Brisbane (Pearson c^2 =46.3, P<0.001).

Association between emphysema severity and reporting in Melbourne (I) (Pearson c^2 =18.3, P=0.019)

Table 5. Coronary artery calcification by grade, by site, as described in clinical reports*

Finding	Brisbane	Melbourne (I)
**Coronary artery calcification**		
Total number of people	595	408
Absent†		
Actionable	1 (0.2%)	4 (1.0%)
Non-actionable	6 (1.0%)	13 (3.2%)
Not reported	168 (28.2%)	129 (31.6%)
Mild†		
Actionable	69 (11.6%)	16 (3.9%)
Non-actionable	170 (28.6%)	140 (34.3%)
Not reported	6 (1.0%)	12 (2.9%)
Moderate†		
Actionable	75 (12.6%)	18 (4.4%)
Non-actionable	39 (6.6%)	53 (13.0%)
Not reported	2 (0.3%)	10 (2.5%)
Severe++		
Actionable	28 (4.7%)	5 (1.2%)
Non-actionable	30 (5.0%)	6 (1.5%)
Not reported	0 (0.0%)	2 (0.5%)

* There were no missing data.

† As categorised in the research checklist.
